# Dual-energy computed tomography in the early evaluation of acute radiation pneumonia

**DOI:** 10.1186/s40001-024-01650-9

**Published:** 2024-02-16

**Authors:** Zong-Sheng Wang, Jing Wu, Gang Yuan, Yong-Bao Liu, Xiao-Ping He

**Affiliations:** 1 Department of Radiology, The First Affiliated Hospital of Kangda College of Nanjing Medical University, The First People’s Hospital of Lianyungang, No. 6 of Zhenhua East Street, Haizhou District, Lianyungang, 222000 Jiangsu China; 2 Department of Oncology, The First Affiliated Hospital of Kangda College of Nanjing Medical University, The First People’s Hospital of Lianyungang, Lianyungang, 222000 Jiangsu China

**Keywords:** Dual-energy, Esophageal cancer, Perfusion imaging, Radiotherapy, X-ray computer

## Abstract

**Objective:**

To investigate the value of dual-energy dual-source computed tomography (DSCT) in evaluating pulmonary perfusion changes before and after radiotherapy for esophageal cancer, and its clinical use in the early diagnosis of acute radiation pneumonia (ARP).

**Methods:**

We selected 45 patients with pathologically confirmed esophageal cancer who received radiotherapy (total irradiation dose of 60 Gy). Dual-energy DSCT scans were performed before and after radiotherapy and the normalized iodine concentrations (NIC) in the lung fields of the areas irradiated with doses of > 20 Gy, 10–20 Gy, 5–10 Gy, and < 5 Gy were measured. We also checked for the occurrence of ARP in the patients, and the differences in NIC values and NIC reduction rates before and after radiotherapy were calculated and statistically analyzed.

**Results:**

A total of 16 of the 45 patients developed ARP. The NIC values in the lung fields of all patients decreased at different degrees after radiotherapy, and the NIC values in the area where ARP developed, decreased significantly. The rate of NIC reduction and incidence rate of ARP increased gradually with the increasing irradiation dose, and the inter-group difference in NIC reduction rate was statistically significant (*P* < 0.05). Based on the receiver operating characteristic (ROC) curve analysis, the areas under the curves of NIC reduction rate versus ARP occurrence in the V_5-10 Gy_, V_10-20 Gy_, and V_> 20 Gy_ groups were 0.780, 0.808, and 0.772, respectively. Sensitivity of diagnosis was 81.3%, 75.0%, and 68.8% and the specificity was 65.5%, 82.8%, and 79.3%, when taking 12.50%, 16.50%, and 26.0% as the diagnostic thresholds, respectively. The difference in NIC values in the lung fields of V_<5 Gy_ before and after radiotherapy was not statistically significant (*P* > 0.05).

**Conclusion:**

The dual-energy DSCT could effectively evaluate pulmonary perfusion changes after radiotherapy for esophageal cancer, and the NIC reduction rate was useful as a reference index to predict ARP and provide further reference for decisions in clinical practice.

## Introduction

With the gradual increase in the incidence of esophageal cancer in China [1, 2] and the widespread use of three-dimensional conformal and intensity-modulated radiotherapy, the incidence of acute radiation pneumonia (ARP) is increasing. As the lung is a radiation-sensitive organ, ARP has become the most serious complication of lung radiotherapy. In the early stages of treatment, ARP is highly reversible [3], but the commonly used X-ray plain film and chest CT are inadequate to help in early diagnosis. Iodine concentration (IC, mg/ml) can be measured with dual-source computed tomography (DSCT) and used to infer the blood supply of the target tissue. We used DSCT to analyze the incidence of ARP and the changes in pulmonary blood perfusion in patients before and after radiotherapy, and we found the following:

## Materials and methods

### Study respondents

#### Profile of respondents

We performed a retrospective analysis of 45 patients with pathologically confirmed esophageal squamous cell carcinoma who received intensity modulated radiation treatment (IMRT) in our institution between January 2017 and December 2018. All patients satisfied the inclusion and exclusion criteria of the study. Among them, 26 patients were male, and 19 patients were female, aged between 49 and 72 years, mean age was 60.96 ± 6.25 years. There were 18 patients with upper esophageal carcinoma, 21 patients with middle esophageal carcinoma, and 6 patients with lower esophageal carcinoma. Among them, 17 patients were in stage T1–2 and 28 patients were in stage T3–4. All of them were treated with three-dimensional conformal intensity-modulated radiation therapy (IMRT) and all successfully completed the treatment course. The study protocol was approved by the Ethics Committee of the hospital (No.: KY-20161130001; Date: 2016-12-30). The scope of the review includes the scientific character and feasibility of the scheme, the design of informed consent and informed consent, the protection of participants' rights and interests, and the protection of their privacy.

### Inclusion criteria for respondents

(1) Patients whose diagnosis was confirmed by clinical pathology.

(2) Those whose Karnofsky score was ≥ 70.

(3) Those who had not undergone radiotherapy or chemotherapy in the past and during the follow-up period after discharge.

(4) Patients who met the following lung radiation dose parameters:

① Lung V_dose_ parameter (percentage of lung volume receiving a certain irradiation dose to the whole lung volume in clinical practice).

40% ≤ V_5 Gy_ ≤ 55%, 20% ≤ V_10 Gy_ ≤ 40%, 10% ≤ V_20 Gy_ ≤ 20%.

② Mean lung dose (MLD) ≤ 13 Gy.

(5) With normal functioning of major organs such as heart, brain, liver, lungs, and kidneys.

(6) With no history of diabetes.

(7) With no history of iodine allergy.

(8) All patients who signed the Informed Consent Form.

(9) Complete all prescribed radiotherapy courses.

### Exclusion criteria

(1) Patients with esophageal cancer in the cervical segment and esophagogastric junction zone.

(2) With an expected survival of < 6 months or with distant metastasis.

(3) Whose body mass index (BMI) was < 18.5 kg/m^2^ or > 30 kg/m^2^.

(4) With thyrotoxicosis or iodine allergy.

(5) With thoracic deformities or the presence of underlying pulmonary disease or pulmonary insufficiency.

(6) With severe cardiac, hepatic, and renal insufficiency.

(7) Patients who did not cooperate with the treatment and related investigations.

### Methods

#### CT scan method

The day before and the day after radiotherapy, the patients underwent a dual-energy mode lung CT enhancement scan from the upper neck to the lower edge of the 12th rib with Siemens Dual Source CT (Somatom Definition Flash; Siemens Medical Solutions, Forchheim, Germany) after lying down for 3 min. Ioversol injection (320 mgI/mL, Jiangsu Hengrui Medicine Co., Ltd.) was chosen as the contrast agent, with an enhancement rate of 3.5 ml/s and the dosage of 1.5 ml/kg. Real-time dynamic exposure dose adjustment (CareDose4D; Siemens Medical Solutions) was used to collect the data. All patients underwent one CT plain scan of the lungs at three months from the start of radiotherapy, and if any adverse findings were present, they were examined as required.

#### Radiotherapy regimen

After conventional localization of the US Varian Unique radiotherapy system, the dual-energy scan data were imported into the radiation treatment planning system (TPS) to determine the target tumor volume (GTV). The clinical target volume (CTV) was 0.5 cm outward from the front to the back and 2.0–3.0 cm outward from the top to the bottom of the GTV, and the planned target volume (PTV) was 0.5 cm outward from the CTV. The radiation treatment planning was evaluated by radiotherapists and physicists and required at least 90% isodose curve wrapping around 95% or more of the PTV; the radical dose of radiotherapy was 60 Gy, with 2 Gy/dose, 1 time/day, 5 times/week; the range of lung radiation dose parameters were set at 40% ≤ V_5 Gy_ ≤ 55%, 20% ≤ V_10_ Gy ≤ 40%, 10% ≤ V_20 Gy_ ≤ 20%, and MLD ≤ 13 Gy.

#### Image and data processing

- 1) Post-processing software

A dual source Syngo VIA workstation with a convolution kernel value of D36f was used to generate 120 kV mixed-energy images after tuning into the Dual-Energy mode, and post-processing was performed using Lung PBV software in CHEST(CA) VNC mode.

- 2) Data measurement and calculation methods

**Fig. 1:**
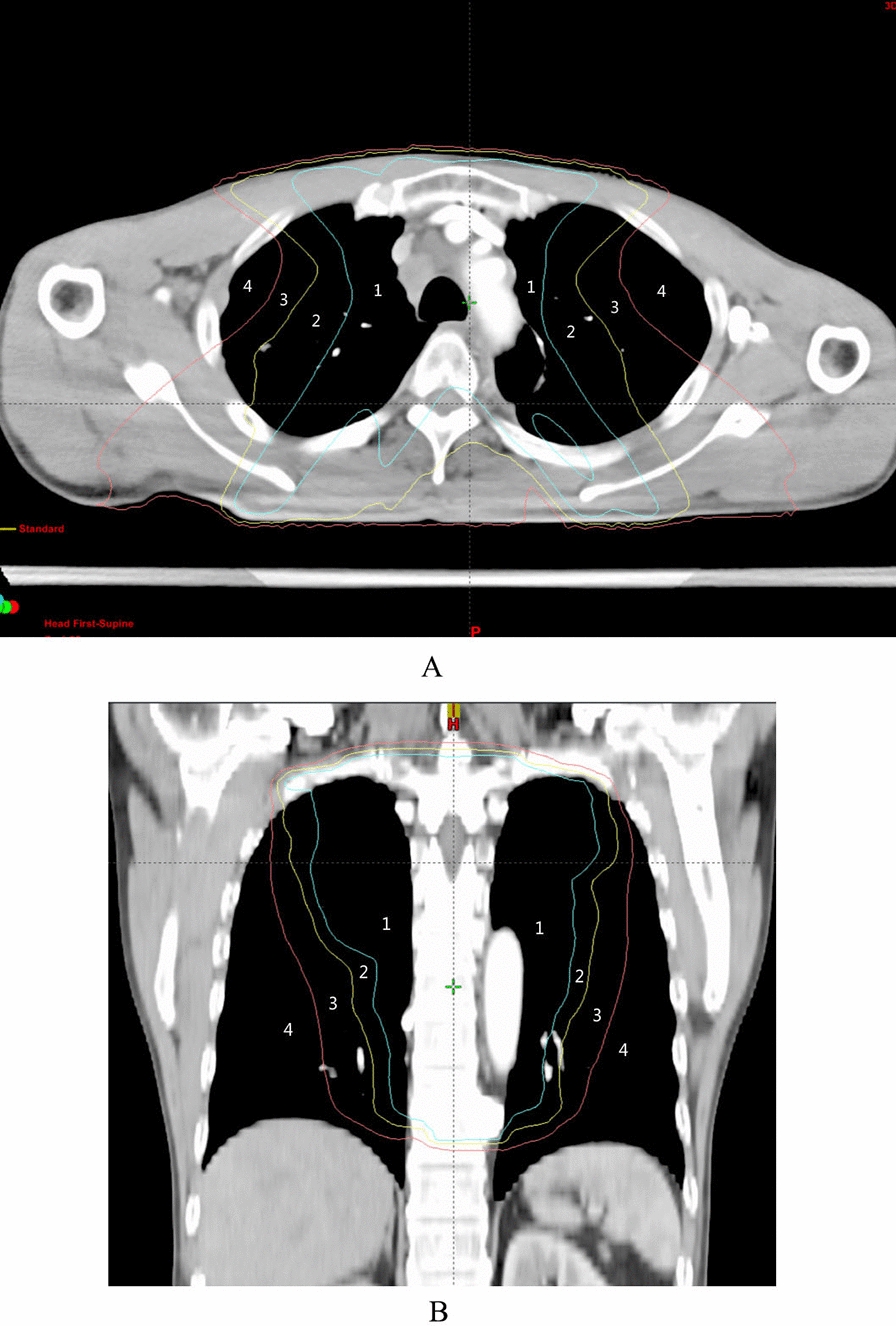
TPS localization map; a shows the transverse localization map of the patient with esophageal cancer; b shows the coronal localization map of another patient, where the green line is the V_20_ dose line, the yellow line is the V_10_ dose line, and the red line is the V_5_ dose line, and the numbered zone 1 is the > 20 Gy dose irradiation range, marked as V_>20 Gy_; zone 2 is the 10–20 Gy dose irradiation range, marked as V_10-20 Gy_; zone 3 is the 5–10 Gy dose irradiation range, marked as V_5-10 Gy_; zone 4 is the < 5 Gy dose irradiation range, marked as V_<5 Gy_

According to the radiation TPS localization map, the V_5 Gy_, V_10 Gy_, and V_20 Gy_ dose line ranges were marked using different color lines by three or more attending physicians in the Imaging Department. The lung tissue ranges that received > 20 Gy, 10–20 Gy, 5–10 Gy, and < 5 Gy irradiation dose coverage individually were determined at the corresponding anatomical levels (set as V_>20 Gy_, V_10-20 Gy_, V_5-10 Gy_, V_<5 Gy_ regions, see Fig. 1a, b for schematic diagrams). The mean iodine concentration values of lung tissue in the regions of V_>20 Gy_, V_10–20 Gy_, V_5–10 Gy_, and V_<5 Gy_ were measured before and after radiation treatment, respectively, ranging from the anterior, middle, and posterior lung fields. NIC values were calculated based on the concentrations of enhanced iodine in the pulmonary arteries of the same level (NIC = Iodine concentration value within the lesion / Iodine concentration value in the pulmonary arteries of the same level), and the left and right lung fields were measured separately.

Region of interest (ROI) selection: The ROI range was set at about 150 mm^2^ for the maximum end-inspiration hold scan, and the average value of iodine concentration at any 3 points of lung tissue in the transverse (coronal) plane in the same region was selected (vertical spacing of any two points ≥ 15 mm), and then the average value of 3 transverse or coronal planes (layer spacing of 2 cm/layer) was calculated as the NIC value of the region. The NIC reduction rate and ARP incidence were calculated relying on the reference localization of the adjacent thicker vessels and bronchi during the measurement.

NIC reduction rate = (NIC parameter value before radiotherapy—NIC parameter value after radiotherapy) ÷ NIC parameter value before radiotherapy × 100%

ARP incidence rate = Number of cases of ARP in the irradiated area ÷ Total number of cases in the irradiated area × 100%

- 3) Comprehensive diagnosis of ARP

The patient was diagnosed with ARP after excluding pulmonary infections and other diseases with reference to the comprehensive clinical diagnostic criteria of the Radiation Therapy Oncology Group (RTOG) [4] along with peripheral blood tests and clinical treatment history. As per RTOG [4] and CT grading criteria [5], we classified the CT manifestations of lung injury into 4 grades according to the imaging signs in the radiation field, margin, or irradiation path: Grade 1: Appearance of more uniform, slightly increased density shadows; Grade 2: Appearance of patchy solid shadows; Grade 3: Appearance of scattered solid shadows; Grade 4: Appearance of dense solid and striated shadows, which corresponded to grades 1–4 in the RTOG grading, respectively [4].

### Statistical analysis

We used SPSS 20.0 software for statistical analysis. Non-numerical data are expressed as rates, and the chi-square test was used for inter-group comparisons. Quantitative data were expressed as $\overline{{\text{x}}}$ ± s, and t-test or analysis of variance for independent samples was used for inter-group comparisons, and paired t-test was used for inter-group comparisons before and after radiotherapy. Pearson’s correlation was used to analyze the correlation between NIC reduction rate and ARP, and receiver operator character (ROC) curve analysis was used to determine the threshold values of NIC reduction rate in V_<5G_, V_5–10G_, V_10–20G_, V_>20G_ groups, and the sensitivity and specificity. 95% confidence interval was used for the reference value, and an α_bilateral_ = 0.05 was used for the test level. *P* < 0.05 was considered a statistically significant difference.

## Results

### Comparative analysis of NIC before and after radiotherapy

We obtained well-structured dual-energy lung scan images with reproducible NIC measurement values for all patients. The mean NIC values were 0.176 ± 0.027 on the left side of the lung field and 0.173 ± 0.049 on the right side for patients before radiotherapy, and the difference was not statistically significant (*t* = 0.325, *P* = 0.747). The inter-group differences in NIC values in the regions of V_<5 Gy_, V_5–10 Gy_, V_10–20 Gy_, and V_>20 Gy_ before radiotherapy were not statistically significant (*F* = 1.410, *P* = 0.240). After radiotherapy, the NIC values in the V_5–10 Gy_, V_10–20 Gy_, and V_>20 Gy_ regions showed a decrease, and the differences were statistically significant (*P* < 0.001) (Table 1). The NIC reduction rate gradually increased with increasing radiotherapy dose, and the average NIC reduction rates in the regions of V_5–10 Gy_, V_10–20 Gy_, and V_>20 Gy_ were 12.84%, 17.22%, and 24.08%, respectively, and the differences were statistically significant (*F* = 5.624, *P* = 0.005).

**Table 1:** Comparative analysis of NIC values before and after radiotherapy in the radiotherapy group

Variables	Before radiotherapy	After radiotherapy	t	P
V_<5G_	0.169 ± 0.030	0.164 ± 0.026	1.974	0.055
V_5-10G_	0.177 ± 0.034	0.151 ± 0.029	7.323	< 0.001
V_10-20G_	0.172 ± 0.026	0.147 ± 0.026	7.684	< 0.001
V_>20G_	0.181 ± 0.029	0.136 ± 0.031	9.592	< 0.001

### Analysis of the ARP incidence and its related factors after radiotherapy

#### Imaging characteristics of ARP after radiotherapy

**Fig. 2:**
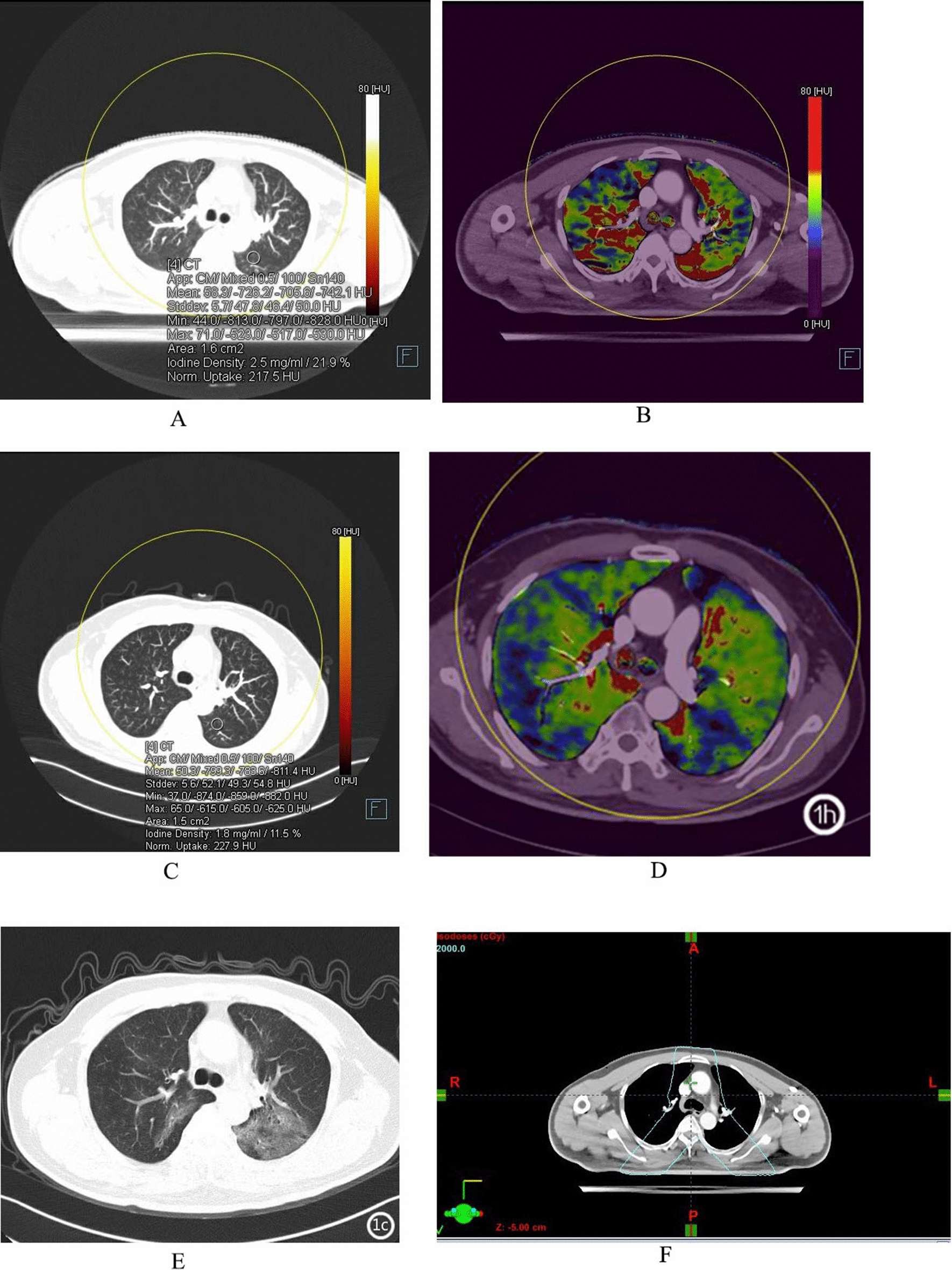
Male/60 years old, upper segment esophageal cancer; **a** Dual-energy lung window image before radiotherapy, the NIC value of the left lung is 0.219; **b** Lung PBV color image before radiotherapy, blood flow (red part) is more abundant in bilateral irradiated fields; **c** Dual-energy lung window image after radiotherapy, the NIC value of the left lung is 0.115; **d** Lung PBV color image after radiotherapy, blood flow (red part) is less than before; **e** CT image during follow-up period, ARP is seen in both lungs; **f** TPS transverse localization image, the region of V_>20 Gy_ irradiation is within the green line, and ARP is seen in the region

**Fig. 3:**
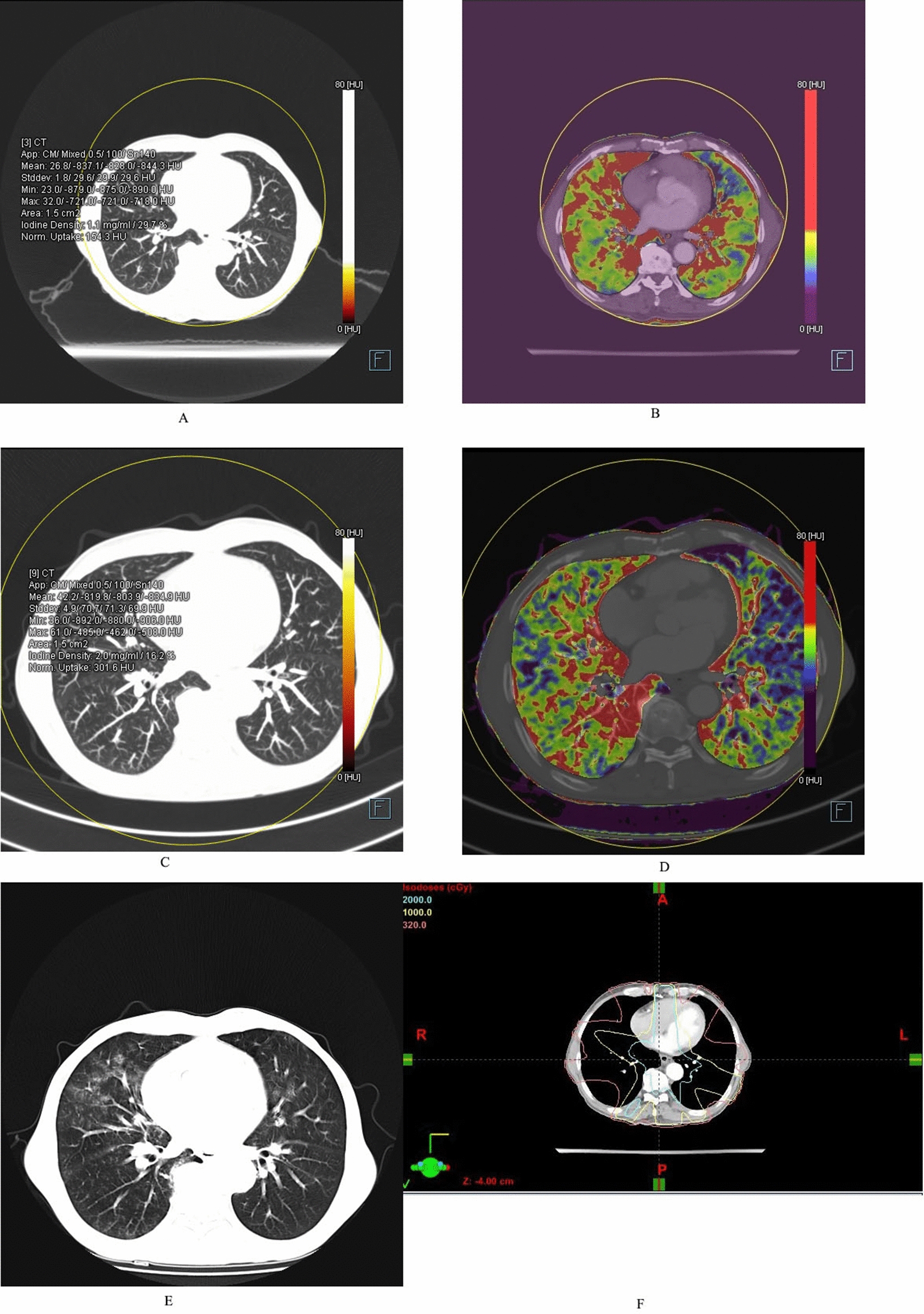
Male/64 years old, middle segment esophageal cancer; **a** Dual-energy lung window image before radiotherapy, the NIC value of the right middle lung is 0.297; **b** Lung PBV color image before radiotherapy, blood flow (red part) is more abundant in bilateral irradiated fields; **c** Dual-energy lung window image after radiotherapy, the NIC value of the right middle lung is 0.162; **d** Lung PBV color image after radiotherapy, blood flow (red part) is less than before; **e** CT image during follow-up period, ARP is seen in both lungs; **f** TPS transverse localization image, the irradiation range was within the red line, and ARP is seen in the irradiation areas of V_> 20 Gy_, V_10-20 Gy_, and V_5-10 Gy_

**Fig. 4:**
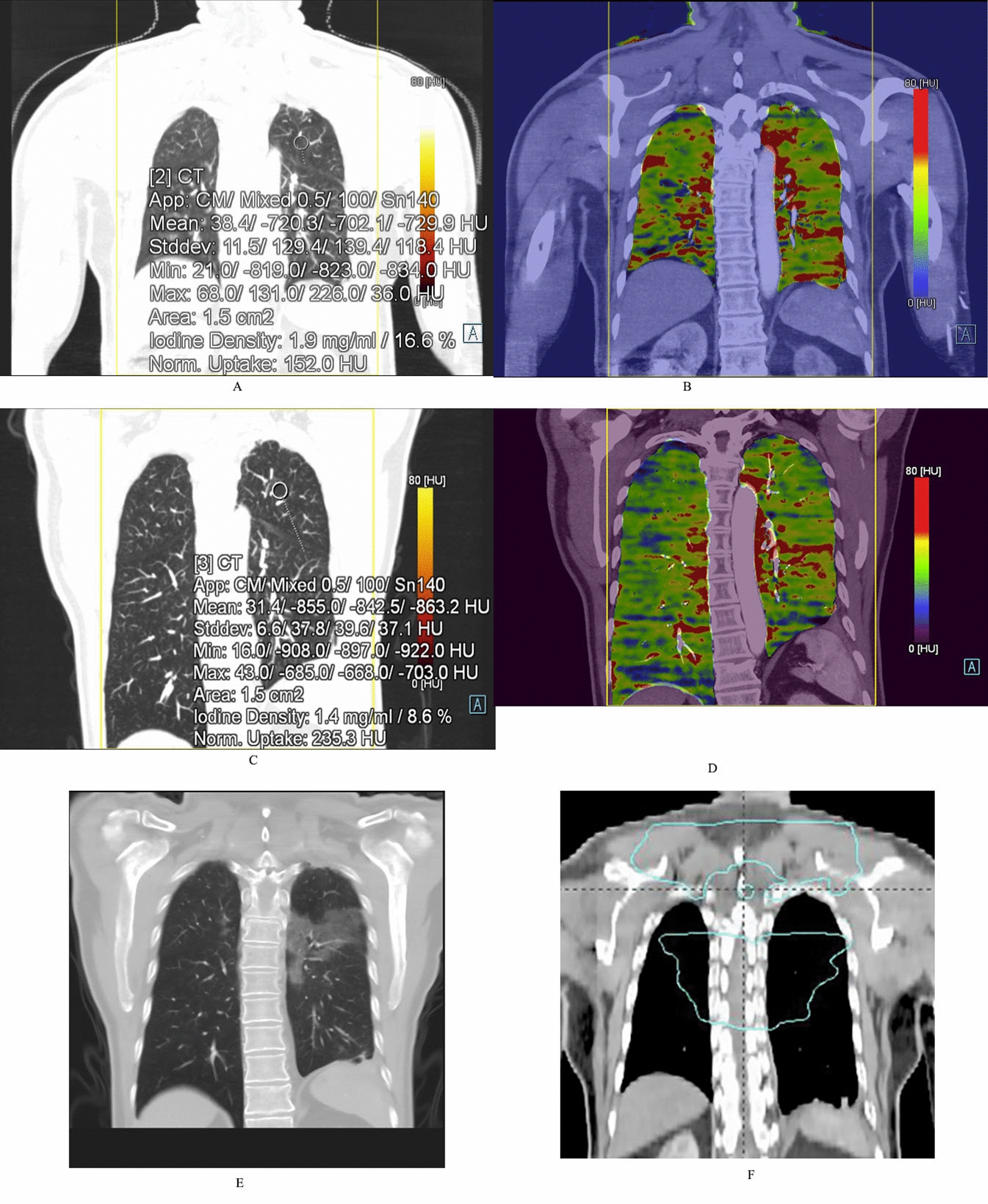
Female/63 years old, upper segment esophageal cancer; **a** Dual-energy lung window image before radiotherapy, the NIC value of the upper left lung is 0.166; **b** Lung PBV color image before radiotherapy, blood flow (red part) is more abundant in bilateral irradiated fields; **c** Dual-energy lung window image after radiotherapy, the NIC value of the upper left lung is 0.086; **d** Lung PBV color image after radiotherapy, blood flow (red part) is less than before. **e** CT image during follow-up period, ARP is seen in both upper lungs; **f** TPS coronal localization image, the region of V_>20 Gy_ irradiation is within the green line, and ARP is seen in the region

From the start of radiotherapy to the end of the 3-month follow-up period, a total of 16 patients were diagnosed with ARP and were classified as the ARP group, while the remaining 29 patients were classified as the non-ARP group. In the ARP group, 2 patients at the end of radiotherapy and 14 patients before the end of the 3-month period showed typical imaging signs in the lungs. Based on the RTOG [4] and CT grading evaluation criteria [5], the main grade 1 CT manifestations were patchy, uniform exudation shadows with slight density increase (11 cases in total), grade 2 manifestations were primarily patchy consolidation shadows (4 cases), and grade 3 manifestations were mostly concurrent exudation and patchy consolidation shadows (1 case). There were a total of 11 patients with grade 1 and 5 patients with grade ≥ 2; ARP with grade ≥ 2 occurred in the V_>20G_ region (5 patients) and the V_10-20G_ region (2 patients). Several patients developed ARP in different irradiation regions at the same time, and the specific occurrence was as follows: 9 patients developed ARP in regions of V_>20 Gy_, V_10–20 Gy_, and V_5–10 Gy_, 3 patients developed ARP in regions of V_>20G_ and V_10-20G_, and 4 patients developed ARP in regions of V_>20 Gy_ alone; no patient developed ARP in regions of V_<5 Gy_. Conventional CT scan showed asymmetric thickening of the esophageal wall in the transverse section, which formed a soft tissue mass with the esophageal wall as the axis, and the lumen of the esophagus was irregularly narrowed. At the end of radiotherapy, two patients showed light flocculent exudative changes in the irradiated lung field, and were finally diagnosed with ARP, and a total of two patients were detected by conventional CT. It was found in dual-energy DSCT examination that at the end of radiotherapy, 16 patients showed significant NIC reduction areas within the irradiated lung fields compared with that before radiotherapy (Figs. 2a, 3, and 4f), and all were confirmed as ARP by CT follow-up. The dual-energy DSCT scan was significantly better than conventional CT in the diagnosis of early ARP.

#### Analysis of the differences between the ARP and non-ARP groups

The differences between the ARP and non-ARP groups of patients were not statistically significant (*P* > 0.05) with respect to the variables of gender, staging of esophageal cancer, lesion location, age, NIC before radiotherapy, V5 Gy, V10 Gy, V20 Gy, and MLD, and the differences were statistically significant (*P* < 0.05) between the groups with respect to NIC values after radiotherapy, and statistically significant (*P* < 0.001) between the groups with respect to NIC reduction rates (Table 2).

**Table 2:** Analysis of differences between the ARP and non-ARP groups

Variables	ARP group (n = 16)	Non-ARP group (n = 29)		
Gender				
Male	9 (56.2%)	17(58.6%)	0.024^#^	1.000
Female	7 (43.8%)	12(41.4%)		
Staging of T in esophageal cancer				
T1-2	6 (37.5%)	11 (37.9%)	0.001^#^	1.000
T3-4	10 (62.5%)	18 (62.1%)		
Lesion location				
Upper	4 (25.0%)	14 (48.3%)	2.801^#^	0.246
Middle	10 (62.5%)	11 (37.9%)		
Lower	2 (12.5%)	4 (13.8%)		
Age	61.13 ± 5.86	60.86 ± 6.55	− 0.134^*^	0.894
NIC before radiotherapy	0.178 ± 0.033	0.173 ± 0.023	− 0.582^*^	0.564
NIC after radiotherapy	0.142 ± 0.023	0.154 ± 0.017	2.035^*^	0.048
NIC reduction rate (%)	19.87 ± 5.70	10.79 ± 3.79	− 6.408^*^	< 0.001
V5 (%)	50.13 ± 2.58	49.70 ± 2.13	− 0.594^*^	0.555
V10 (%)	35.60 ± 2.77	34.61 ± 2.05	− 1.367^*^	0.179
V20 (%)	15.44 ± 2.70	14.51 ± 2.08	− 1.291^*^	0.204
MLD (G)	11.64 ± 1.10	11.11 ± 0.75	− 1.920^*^	0.062

^*^Represents t-value, # represents χ^2^ value

#### Analysis of NIC reduction rate and ARP occurrence in regions irradiated with different doses

The occurrence of ARP in different dose irradiation regions was as follows: 16 patients developed ARP in the V_>20G_ region, with the incidence rate of 35.56%, and this included 11 patients with grade 1 and 5 patients with grade ≥ 2; 12 patients developed ARP in the V_10–20 Gy_ region, with the incidence rate of 26.67%, consisting of 10 patients with grade 1 and 2 patients with grade ≥ 2; 9 patients developed ARP in the V_5–10 Gy_ region, with the incidence rate of 20.0%, all with grade 1; none of the patients developed ARP in the V_<5 Gy_ region. The NIC reduction rate and ARP incidence in different dose irradiation regions were strongly correlated, and the correlation coefficients between NIC reduction rate and ARP incidence in V_5–10 Gy_, V_10–20 Gy_, and V_>20 Gy_ groups were r_5–10 Gy_ = 0.530, *P* < 0.001; r_10–20 Gy_ = 0.588, *P* < 0.001; *r*_>20 Gy_ = 0.492, *P* = 0.001, respectively.

In the ARP group, the mean NIC reduction rate was 16.92 ± 5.28% in patients with ARP of grade 1 and 24.40 ± 3.85% in patients with ARP of grade ≥ 2, and the inter-group difference was statistically significant (*t* = -2.480, *P* = 0.026).

### ROC curve analysis of NIC reduction rate on ARP diagnostic efficacy in regions irradiated with different doses

**Fig. 5:**
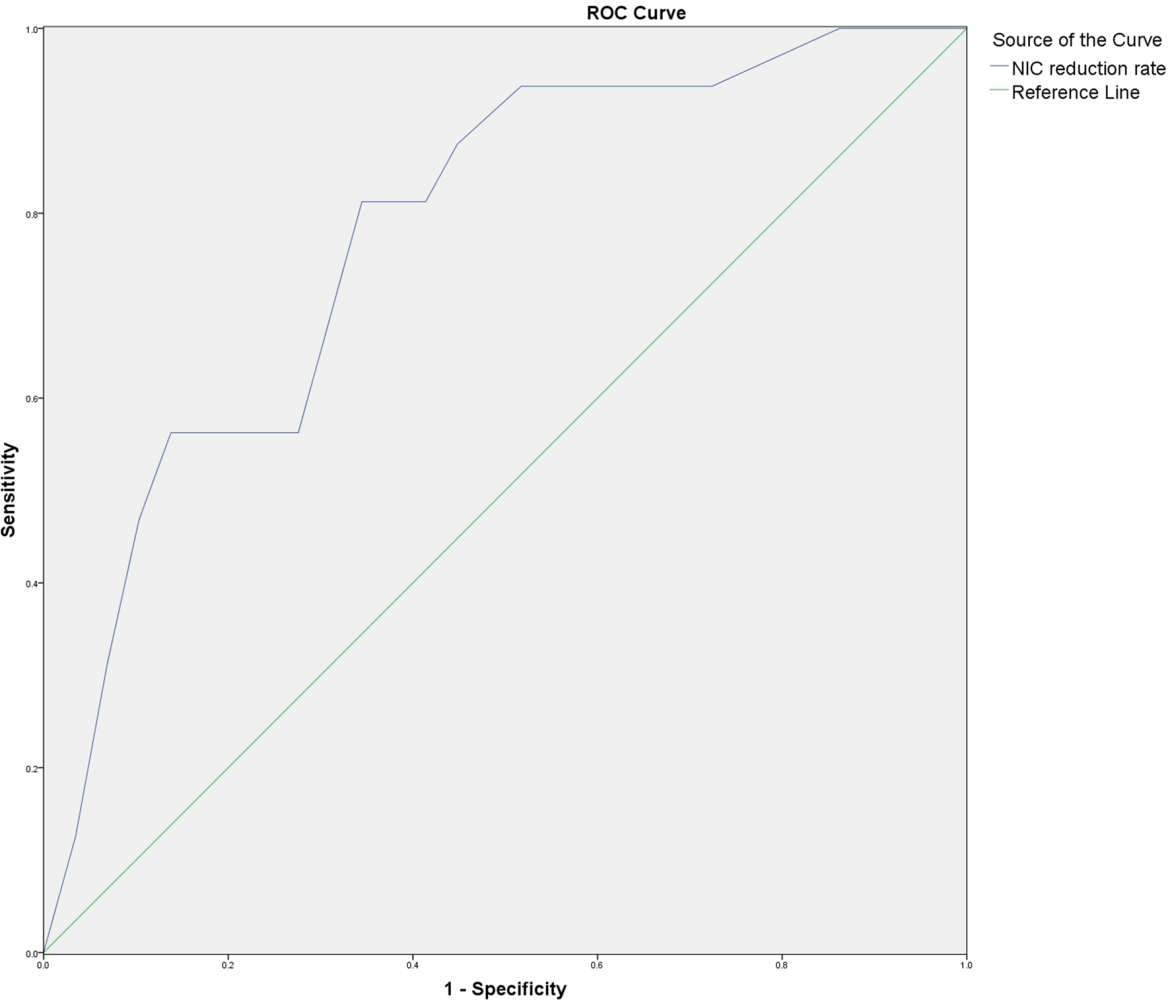
ROC curve of NIC reduction rate in V_5-10 Gy_ group. Shows the ROC curve of NIC reduction rate for patients in the V_5-10 Gy_ group, and the area under the curve is 0.780

**Fig. 6:**
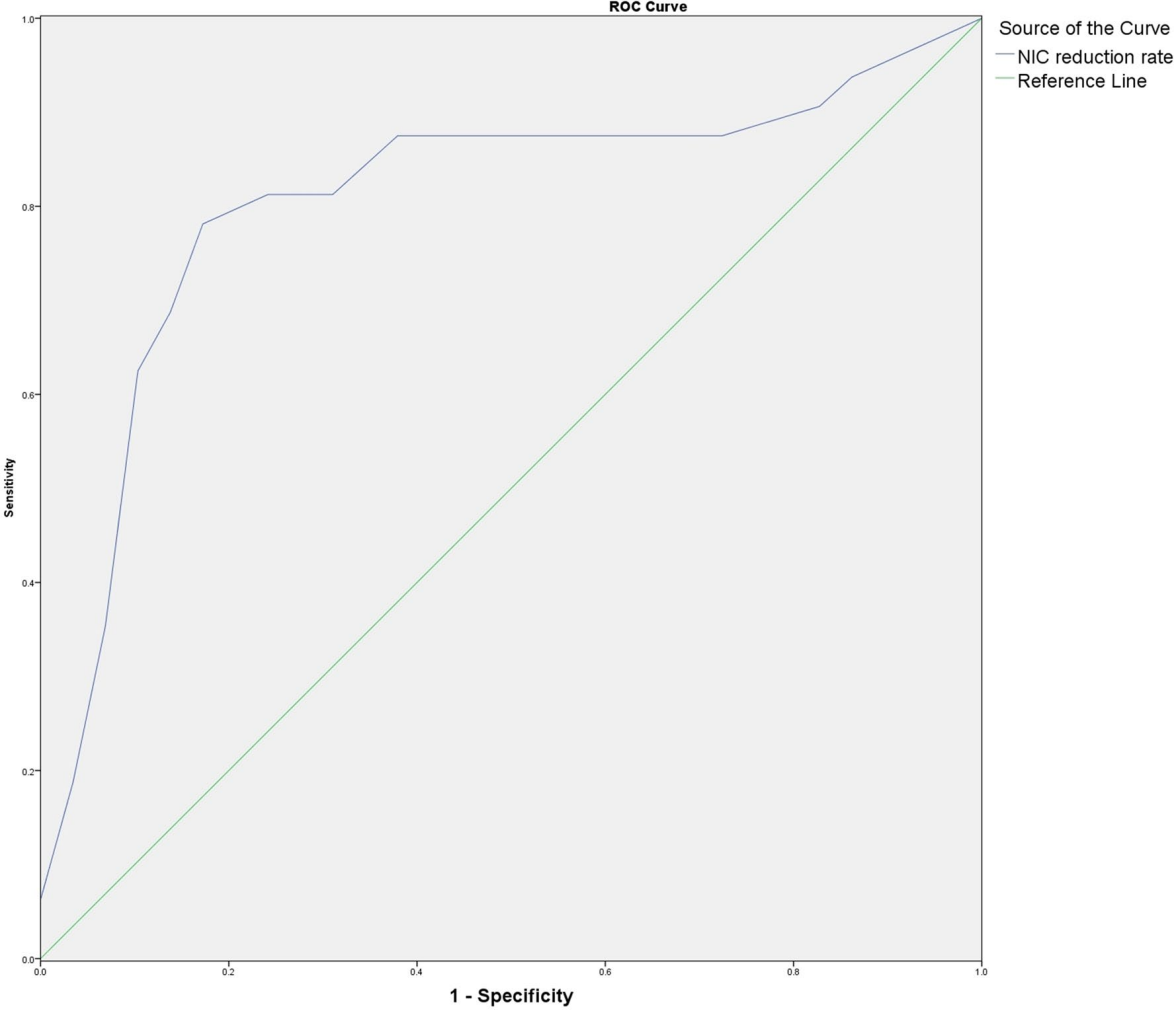
ROC curve of NIC reduction rate in V_10-20 Gy_ group. Shows the ROC curve of NIC reduction rate for patients in the V_10-20 Gy_ group, and the area under the curve is 0.808

**Fig. 7:**
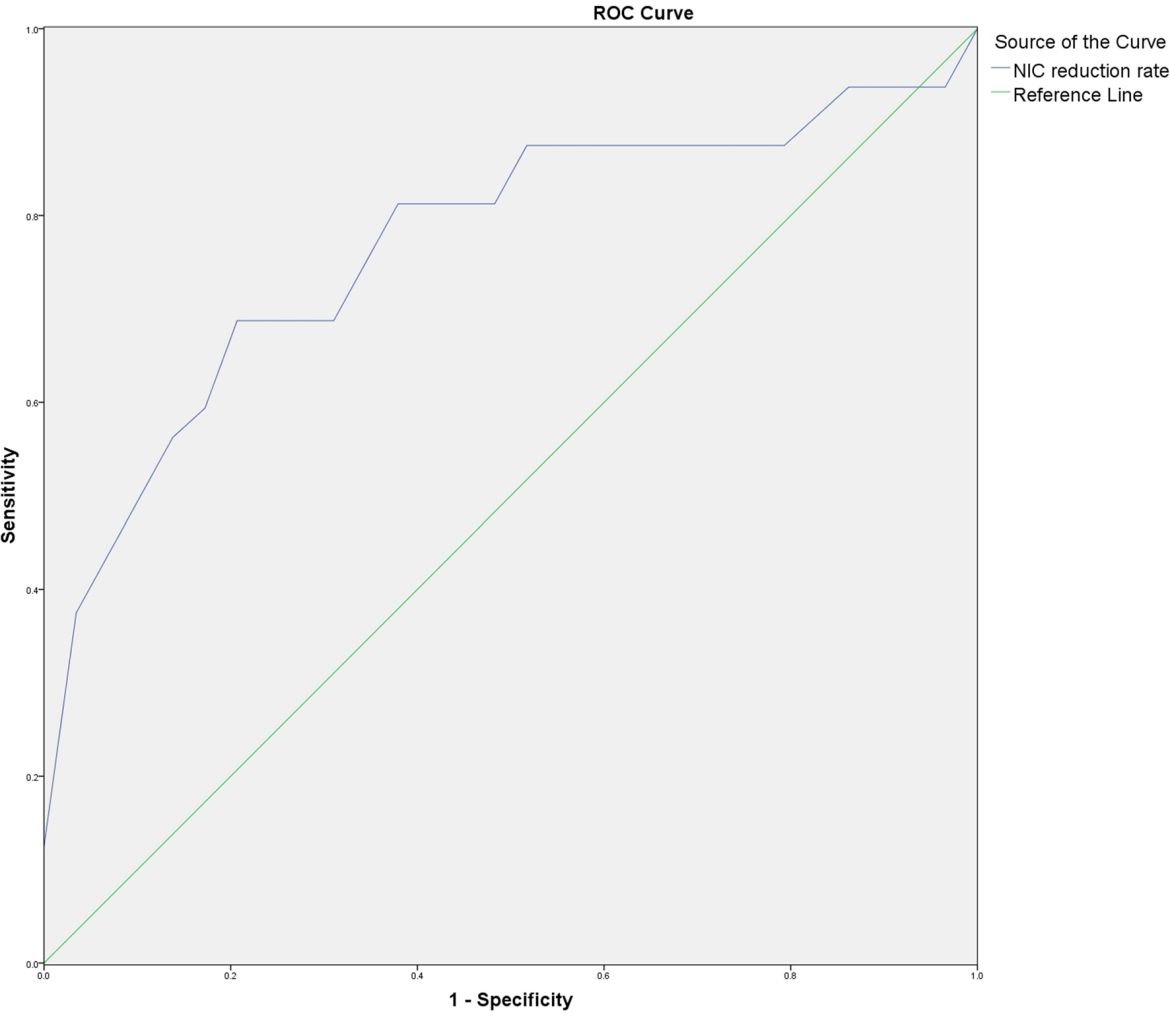
ROC curve of NIC reduction rate in V_>20 Gy_ group. Shows the ROC curve of NIC reduction rate for patients in the V_> 20 Gy_ group, and the area under the curve is 0.772

The NIC values of lung tissues were measured before and after radiotherapy in V_5-10G_, V_10-20G_, and V_>20G_ groups. ARP occurred in all groups when the NIC reduction rate was used as the observation index, and the ROC curve analysis was done in V_5–10 Gy_, V_10–20 Gy_, and V_>20 Gy_ groups based on the NIC reduction rate and the occurrence of ARP after radiotherapy. According to the ROC curve (Figs. 5, 6, and 7), with 12.50% as the diagnostic threshold, the area under the curve of NIC reduction rate versus ARP occurrence in the V_5–10 Gy_ group was 0.780, and the sensitivity and specificity were 81.3% and 65.5%, respectively.NIC reduction rate of V5-10 Gy group 95% confidence interval is 0.641–0.919. With 16.50% as the diagnostic threshold, the area under the curve of NIC reduction rate versus ARP occurrence in the V_10–20 Gy_ group was 0.808, and the sensitivity and specificity were 75.0% and 82.8%, respectively.NIC reduction rate of V10-20 Gy group 95% confidence interval is 0.656 to 0.960. With 26.0% as the diagnostic threshold, the area under the curve of NIC reduction rate versus ARP occurrence in the V_>20G_ group was 0.772, and the sensitivity and specificity were 68.8% and 79.3%, respectively.NIC reduction rate of V > 20 Gy group 95% confidence interval is 0.613–0.931. In summary, the NIC reduction rate could be used as an effective index to predict the occurrence of ARP (Table 3).

**Table 3:** Results of ROC curve analysis of NIC reduction rate versus ARP incidence

Variables	*Az*	*P*	Threshold (%)	Sensitivity (%)	Specificity (%)
NIC reduction rate of V_5–10 Gy_ group	0.780	0.002	12.50	81.3	65.5
NIC reduction rate of V_10–20 Gy_ group	0.808	0.001	16.50	75.0	82.8
NIC reduction rate of V_>20 Gy_ group	0.772	0.003	26.0	68.8	79.3

## Discussion

Acute radiation pneumonia (ARP) refers to early radiation-induced lung injury (RILI) that occurs within 1–3 months after radiotherapy; the incidence of RILI is reported to be about 5%–58% in China and abroad, of which the incidence of ARP is about 13%–37%, and the incidence of symptomatic ARP (≥ grade 2) is about 1–10% [6, 7]. ARP over grade 2 can affect the health and quality of life of patients once it occurs; it may then lead to severe pulmonary fibrosis, impair respiratory function, and even result in life-threatening respiratory failure.

Radiation injury to the lungs manifests very early on as a pathological change in ARP. When everything is working as it should, the lung is not only kept relatively dry, which is helpful for gas exchange, but it also has the right amount of water, which is conducive to the survival and metabolism of tissue cells, thanks to the lung's rich microvascular network, alveolar-capillary permeability, lymphatic vessels, and the fluid between tissues being maintained in dynamic balance. Radiation therapy initially results in local vascular endothelial cell injury, followed by secondary diffuse alveolar injury and subsequent alterations in congestion, edema, and exudation. An rise in both interstitial pressure (the pressure between the alveolar wall and the capillary wall) and alveolar pressure causes alterations in the microcirculation of the alveoli and, consequently, a disruption in blood perfusion. When the irradiation dose increases, the damage worsens. According to statistics, the irradiation dose of 1 Gy can reduce the ratio of local lung perfusion to the average count of the whole lung in normal lung tissue by 0.67% [8].With the extension of irradiation time and scope, the lung injury process of patients was repeated and gradually aggravated, and a series of clinical symptoms appeared. CT began to show increased density of the irradiated lung, and eventually ARP occurred.

ARP is different from general bacterial lung infections in that early treatment is highly reversible and early identification is critical [3]. Conventional imaging signs usually appear 4 weeks or even later after chest radiotherapy, which lags clinical symptoms by about several weeks. Commonly used clinical X-ray plain films and chest CT (including high-resolution CT) are currently inadequate for the timely detection of ARP with grade ≤ 2, and this causes delay in treatment. The use of various imaging methods such as SPECT and magnetic resonance spectroscopy techniques to predict RILI is a new research hotspot in China and abroad, however, relevant research in this field is still in its infancy and further exploration is still needed [9–15].

The DSCT used in this study was manufactured by Siemens, Germany, has two independent sets of X-ray tubes (140 kV for tube A and 100 kV for tube B) and corresponding data acquisition systems, which can be used to obtain dual-energy images through post-processing. DSCT can quantitatively reflect the tissue blood supply to the lesion by measuring the iodine concentration (IC, mg/ml). It is used extensively clinically [15]. The concept of normalized iodine concentration (NIC) was introduced to eliminate individual differences such as patient weight, blood volume, and the flow rate of contrast agent in vivo. Since the lung contains a rich capillary network, the Lung Perfusion Blood Volume (Lung PBV) Imaging software in the dual-energy examination mode of DSCT can display the distribution of iodine in target organs and the corresponding anatomical information and can accurately measure the iodine concentration values in local lung tissue, thus quantitatively reflecting the blood perfusion changes in lung tissue before and after radiotherapy.

Lung is a dual blood supply organ, and pulmonary blood flow is normally provided by both the pulmonary artery and the bronchial artery under normal circumstances, however, bronchial artery blood flow accounts for only about 5%, while pulmonary artery blood supply accounts for more than 95%, so normal pulmonary perfusion is determined mainly by the amount of pulmonary artery blood flow. Several studies have found that pulmonary blood distribution is influenced by both gravity and the structure of the pulmonary vascular tree, with the presence of a perfusion gradient in the direction of gravity, which is also the main influencing factor. In our study, in order to avoid the effect of gravity on pulmonary blood flow values as much as possible, the patient was asked to lie flat for 3 min before the examination, the ROI size was set at 150 mm^2^, the average NIC value was obtained by multi-point and multi-planar measurements, and the changes in blood perfusion in irradiated lung fields after radiotherapy were assessed by calculating the NIC reduction rate. In the selection of radiotherapy group cases, we set the lower limit of Vdose parameter for patients, which required 40% ≤ V5 ≤ 55%, 20% ≤ V10 ≤ 40%, and 10% ≤ V20 ≤ 20%, aiming to ensure sufficient coverage of the lung field area irradiated with different doses, include sufficient anterior, middle, and posterior lung fields to facilitate multi-point vertical measurement, reduce the influence of gravity, and improve the accuracy of the measurement.

By the end of follow-up, the ARP incidence in the radiotherapy group of this study was 35.56% (16/45 cases), with the incidence of ARP of grade ≥ 2 being 11.1% (5/45 cases), and this finding was comparable to that reported in literature [6, 7]. ARP is influenced by many factors and is the result of a combination of pathogenic factors, among which radiotherapy, especially the Vdose parameter, is the main influencing factor. The inclusion criteria, radiotherapy dose, V5, V10, V20, and MLD settings of the selected patients undergoing radiotherapy for esophageal cancer in this study were consistent with the findings of most scholars [16, 17]. The aim of the IMRT selected for the radiotherapy regimen was to assess the effect of changes in pulmonary perfusion on the incidence of ARP under the least interfering factors. The results demonstrated that the NIC values of lung fields in all patients showed different degrees of decrease after radiotherapy, and the NIC reduction rate of lung tissue in the area where ARP occurred was significantly greater than that in the area where ARP did not occur, which indicated that the pulmonary perfusion impairment was more obvious in this area. With the increase of irradiation dose, the NIC reduction rate and ARP incidence in the area irradiated with < 5 Gy, 5–10 Gy, 10–20 Gy, and > 20 Gy doses gradually increased, and the NIC reduction rate of those who developed ARP of grade ≥ 2 or higher was significantly higher than that of patients who developed ARP of grade 1, and the inter-group differences were statistically significant (*P* < 0.05). This indicated that the degree of pulmonary perfusion impairment progressively increased with increasing dose of irradiation and was correlated with the incidence and grade of occurrence of ARP. The decrease of pulmonary perfusion in the areas more sensitive to radiation was significantly greater than that in the areas that were non-sensitive. During radiotherapy, if significant NIC reduction of local lung tissues is found, the risk of ARP must be considered. Since the sensitivity of lung tissues to radiation differs in different areas, when designing the radiotherapy plan, the radiation should be made to pass through the non-sensitive areas as much as possible to reduce lung impairment.

In this study, we analyzed the early diagnostic value of NIC reduction rate on ARP using the ROC curve, and the results demonstrated that the areas under the curves of NIC reduction rate versus ARP occurrence in V_5–10 Gy_, V_10–20 Gy_, and V_>20 Gy_ groups were 0.780, 0.808, and 0.772, respectively, and the sensitivity of diagnosis were 81.3%, 75.0%, and 68.8%, respectively, with 12.50%, 16.50%, and 26.0% as the diagnostic thresholds; and the specificity were 65.5%, 82.8%, and 79.3%, respectively. This suggests that the quantitative analysis of the degree of pulmonary perfusion impairment, i.e., the NIC reduction rate, can provide a reference for early diagnosis of ARP. We found that no patient developed ARP in the area irradiated by < 5 Gy dose, and the difference of the NIC values before and after radiotherapy was not statistically significant. This was considered to be related to the fact that the lung tissue coverage in the radiotherapy group receiving < 5 Gy dose was larger (≥ 45%), and the radiation dose of a single radiotherapy was smaller, the duration was longer (30 times), and the body had enough time for compensatory repair. Only when the degree of lung impairment caused by radiation exceeds the body's equilibrium repair capacity, and lung tissue necrosis and fibrosis appear, the lesion can be detected by conventional CT. Since dual-energy DSCT examination has the advantages of easy operation and the ability to provide morphological and functional information of lung tissue at the same time, it can detect and quantitatively assess the degree of lung perfusion impairment at an early stage and is significantly superior to conventional CT in the early diagnosis of ARP.

### Limitation

There are some shortcomings with this study: ① There are several factors that can affect the occurrence of ARP, and the research sample size is inadequate. To determine if the contributions of several factors have a synergistic effect, it is necessary to conduct studies with sizable sample sizes. ② Ionizing radiation dose influence could only be partially explained by measuring NIC values in the pulmonary field in the irradiated areas receiving 5G, 5–10G, 10–20G, and > 20G, even though the boundaries were delimited according to the iso-dose lines of V5, V10, and V20, which are the most widely used in clinical practice. ③ Gravity and the pulmonary vascular tree's structure influenced how blood flowed through the lungs. Even with the use of supine post-lying examinations, multi-layer and vertical multipoint measurements, and NIC reduction rate indicators, it was still possible for interference to occur.

## Conclusion

In conclusion, as a new CT examination method, dual-energy DSCT imaging can effectively evaluate the changes of local lung tissue blood perfusion before and after radiotherapy. It can detect the changes of NIC value earlier, it is more reliable than imaging morphological changes for ARP, and the NIC reduction rate can be used as an effective monitoring index for early prediction of ARP. It thus can assist in developing the clinical plan for radiotherapy.

## Data Availability

The datasets used and/or analysed during the current study available from the corresponding author on reasonable request.

## Abbreviations

DSCT: Dual-energy dual-source computed tomography
ARP: Acute radiation pneumonia
NIC: Normalized iodine concentrations
ROC: Receiver operating characteristic
IMRT: Intensity modulated radiation treatment
TPS: Treatment planning system
GTV: Target tumor volume
CTV: Clinical target volume
PTV: Planned target volume
RILI: Radiation-induced lung injury
NIC: Normalized iodine concentration
